# Novel Evidence of the Increase in Angiogenic Factor Plasma Levels after Lineage-Negative Stem/Progenitor Cell Intracoronary Infusion in Patients with Acute Myocardial Infarction

**DOI:** 10.3390/ijms20133330

**Published:** 2019-07-06

**Authors:** Bartłomiej Baumert, Krzysztof Przybycień, Edyta Paczkowska, Maciej Kotowski, Ewa Pius-Sadowska, Krzysztof Safranow, Jarosław Peregud-Pogorzelski, Zdzisława Kornacewicz-Jach, Małgorzata Peregud-Pogorzelska, Bogusław Machaliński

**Affiliations:** 1 Department of General Pathology, Pomeranian Medical University, 71-111 Szczecin, Poland; 2 Department of Cardiology, Pomeranian Medical University, 71-111 Szczecin, Poland; 3 Department of Biochemistry and Medical Chemistry, Pomeranian Medical University, 71-111 Szczecin, Poland; 4 Department of Paediatric Oncology, Pomeranian Medical University, 71-252 Szczecin, Poland

**Keywords:** angiogenic trophic factors, neurotrophins, cell therapy, myocardial infarction, stem cells

## Abstract

Cell therapy raises hope to reduce the harmful effects of acute myocardial ischemia. Stem and progenitor cells (SPCs) may be a valuable source of trophic factors. In this study, we assessed the plasma levels of selected trophic factors in patients undergoing application of autologous bone marrow (BM)-derived, lineage-negative (Lin^−^) stem/progenitor cells into the coronary artery in the acute phase of myocardial infarction. The study group consisted of 15 patients with acute myocardial infarction (AMI) who underwent percutaneous revascularization and, afterwards, Lin^−^ stem/progenitor cell administration into the infarct-related artery. The control group consisted of 19 patients. BM Lin^−^ cells were isolated using immunomagnetic methods. Peripheral blood was collected on day 0, 2, 4, and 7 and after the first and third month to assess the concentration of selected trophic factors using multiplex fluorescent bead-based immunoassays. We found in the Lin^−^ group that several angiogenic trophic factors (vascular endothelial growth factor, Angiopoietin-1, basic fibroblast growth factor, platelet-derived growth factor-aa) plasma level significantly increased to the 4th day after myocardial infarction. In parallel, we noticed a tendency where the plasma levels of the brain-derived neurotrophic factor were increased in the Lin^–^ group. The obtained results suggest that the administered SPCs may be a valuable source of angiogenic trophic factors for damaged myocardium, although this observation requires further in-depth studies.

## 1. Introduction

Significant progress in the interventional treatment of patients with acute coronary syndromes (ACS) has contributed to the substantial improvement of prognosis. However, the consequences of the loss of myocardium due to myocardium infarction leads to chronic heart failure. Contemporary data show that the incidence of ST-elevation myocardial infarction (STEMI) in European countries ranges from 43–144 per 100,000 per year [1]. Although survival of patient with STEMI has been improved with optimal medical therapy, registries indicate hospital mortality varies between 4%–12%, while one-year mortality among STEMI patients in the angiography register is approximately 10% [2]. To date, cell-based therapy applied by the intracoronary route has been tested in acute coronary syndromes as adjuvant therapy [3,4]. Its efficacy seems to be limited because of a number of factors.

Stem cell-based methods of treatment can be safely and efficiently employed for regeneration of damaged vascularized organs. However, clinical trials conducted so far with the use of cell therapy in cardiac repair and regeneration in chronic heart failure have yielded neutral or, at most, marginally positive outcomes [5,6]. Other stem cell-based clinical trials in cardiovascular therapies have focused on treatment immediately after injury among STEMI patients [7,8,9,10,11]. The mechanisms for stem cell-based therapies of the heart diseases are complex [12]. The initial understanding of cardiac tissue repair involved direct replacement of damaged cardiomyocytes by transplanted stem cells through differentiation processes. Differentiated cardiac stem cells were expected to repair the host tissue by direct tissue replacement [6,13]. However, many observations of very limited stem cell engraftment and direct differentiation of injected cells into cardiomyocytes and vascular cells, either by transdifferentiation or cell fusion, did not comprehensively explain the cardiac benefits [13]. Later on, secretion of growth factors by SPCs was considered as the major contributor to the functional benefits of stem cell-based therapies [14]. It is important that, besides the potential to self-renew and differentiate into mature cells, SPCs exert trophic, anti-apoptotic, and angiopoietic activity by producing important growth factors [15,16]. Among them are neurotrophic factors such as brain-derived neurotrophic factor (BDNF) and glial-derived neurotrophic factor (GDNF) as well as angiopoietic factors such as vascular endothelial growth factor (VEGF) and hepatocyte growth factor (HGF) [15]. In the context of heart tissue regeneration and protection, insulin-like growth factor-1 (IGF-1) could inhibit apoptosis of cardiomyocytes, recruit endogenous stem cells, and promote angiogenesis [17]. Other factors released by SPCs such as VEGF, fibroblast growth factor (FGF) [18], angiopoietin [19] augment angiogenesis. Thus, adult stem cells derived mostly from the bone marrow (BM) or adipose tissue that express a high-secretory profile seem to hold great promise [6]. In our previous work, by employing reverse transcription–polymerase chain reaction, we found that BM-derived stem/progenitor CD34^+^ cells expressed several mRNA transcripts for neurotrophins, and subsequently we confirmed their presence in cells at the protein level by employing immunofluorescence staining [20]. Selected BM-derived Lin^−^ cells consist of a heterogeneous SPC population that contains a small percentage of stem cells. Most of the cells are progenitors. A methodology based on immunomagnetic negative selection has been developed to deplete mononuclear cells (MNCs) of hematopoietic lineage, marker-expressing mature cells. We have previously shown that umbilical cord blood (UCB)-derived SPCs, especially Lin^−^ cells, strongly and specifically express classical neurotrophins (NTs) and the novel neurotrophic cytokines as well as VEGF. We have also shown that these secreted factors support neuronal proliferation and in vitro survival in a conditioned medium from Lin^−^ SPCs [15]. Interestingly, it has been reported that cell-to-cell contact was pivotal to the functional benefits of cell therapies [21].

Growth factors promote survival of cardiomyocytes and stimulate angiogenesis, leading to protection against ischemia and the decrease of heart remodeling [22,23,24,25,26]. Beneficial effects of SPCs might be explained by paracrine and trophic effects of growth as well as chemotactic factors (i.e., cytokines that are released by the cells). Intracoronary application of autologous SPCs was confirmed as a safe and feasible method. To our knowledge, neurotrophins and angiogenic factor concentration in the blood of STEMI patients during the course of experimental Lin^−^ cell therapy have not been studied comprehensively so far. In this study, we aimed to investigate whether intracoronary injection of autologous BM-derived Lin^−^ cells was safe in STEMI patients and whether it could lead to the improvement of left ventricular ejection fraction. Because growth factors play a pivotal role in regeneration, and SPCs can exert a number of growth factors, we hypothesized that adjuvant cell therapy could also bring specific changes in various neurotrophins, angiogenic factors, and other factor profiles in blood plasma of STEMI patients.

## 2. Results

### 2.1. Baseline Characteristics

Table 1 shows patient baseline characteristics. The study population consisted of males, and the median age (IQR) was 54.5 (11.3) y in Lin^−^ group and 52 (11.5) y in control group. 

Most patients (71%) had anterior STEMI due to occlusion of left anterior descending coronary artery (LAD), and the median time from onset of chest pain to reperfusion therapy was 4 (3.75) h for Lin^−^ and 3.5 (5.75) h for the control group. In each patient, during the procedure, only the infarct-related artery was supplied. In Lin^−^ group, 10 patients had LAD, 2 patients had left circumflex artery (LCX), and 3 patients had right coronary artery (RCA) supplied. In the control group, 14 patients had LAD, 1 patient had LCX, and 4 patients had RCA supplied.

Patients in the control group had a lower ejection fraction (EF) (median 35% (3.75%) vs. 40% (5.75%), *p* = 0.047), higher left ventricular end-diastolic volume (LVEDV) (median 151.5 (31.75) mL vs. 128.5 (39.25) mL, *p* = 0.025), and higher aortic bulb dimension (Ao) (median 36.5 (6.0) mm vs. 33 (4.75) mm, *p* = 0.043) at the day 0; higher left ventricular end-systolic volume (LVESV) (median 90.5 (16.0) mL vs. 74 (22.25) mL, *p* = 0.043) and higher left ventricular internal dimension at end-diastole (LVIDD) (median 56 (4.0) mm vs. 51 (4.0) mm, *p* = 0.007) at day 0 and 1. A trend toward a lower body mass index (BMI) (*p* = 0.096) and higher left atrium (LA) diameter (*p* = 0.066) in the control group was observed. These differences could be partially explained by the fact that the study was not randomized. Apart from this, there was no statistically significant difference in baseline characteristics (for patient age, time from onset of chest pain to reperfusion therapy, and supplied coronary artery) between Lin^−^ and the control group.

Laboratory tests were performed on the obtained peripheral blood samples, and the selected results are presented in Table 1. Pharmacological treatment was initiated according to current guidelines shortly after primary percutaneous coronary intervention (PCI). In the Lin^−^ group, a mean of 8.37 ± 7.8 × 10^6^ autologous BM-derived Lin^−^ cells were infused in the infarct-related artery the next day after PCI.

### 2.2. Application of Lin^−^ Cells into the Infarct-Related Coronary Artery

In our previous studies, we thoroughly characterized Lin^−^ cells as a heterogeneous population, which consists of precursors, progenitors, and stem cells and lacks mature blood cells [15]. The phenotypic characterization of administered Lin^−^ cells is shown in Table 2. Of note, inter-individual variability related to the number of Lin^−^ cells isolated from the bone marrow from each STEMI patient and subsequently delivered into infarct-related artery underlines the heterogeneity of this study’s patient group.

We did not observe any adverse effects of autologous BM-derived Lin^−^ cells delivered intracoronarily, which makes them a safe and feasible source of material for use in the myocardium for trophic support provision. There were no deaths, transplant-related infections, acute kidney injuries, and no cases of subsequent AMI during one-year follow-up. All patients included in the study in the first day and after 6 and 12 months underwent a Holter ECG for 24 h. At follow-up, no evidence of ventricular or supraventricular arrhythmias in 24 h ECG monitoring was noted.

In all patients, echocardiography was performed by the same professional blinded to the treatment arm on the day of admission, and subsequent echocardiographic examinations were carried out in all patients on day 0, 2, 4, and 7 as well as 1, 3, 6, and 12 months after myocardial infarction. We analyzed the results obtained by ultrasonography and compared them between groups. The EF, LVEDV, and LVESV parameters evaluated in subsequent time intervals during one-year follow-up did not significantly differ between the groups. Interestingly, at 6 and 12 months of observation, the heart ventricles in the control group began to undergo unfavorable reconstruction by increasing their diameter (median (IQR); month 6—55.5 (10.25) mm vs. 49.5 (9.75) mm, *p* = 0.05; month 12—58 (9.0) mm vs. 53 (9.5) mm, *p* = 0.05). Similarly, the diameter of the aortic bulb tended to increase in the control group at 6 (median; 37 (5.5) mm vs. 35.5 (5.0) mm, *p* = 0.066) and 12 months (median; 35 (5.0) mm vs. 33 (5.75) mm, *p* = 0.071) (Table 3).

### 2.3. Brain-Derived Neurotrophic Factor (BDNF) and Glial-Derived Neurotrophic Factor (GDNF) Plasma Levels

**Figure 1:**
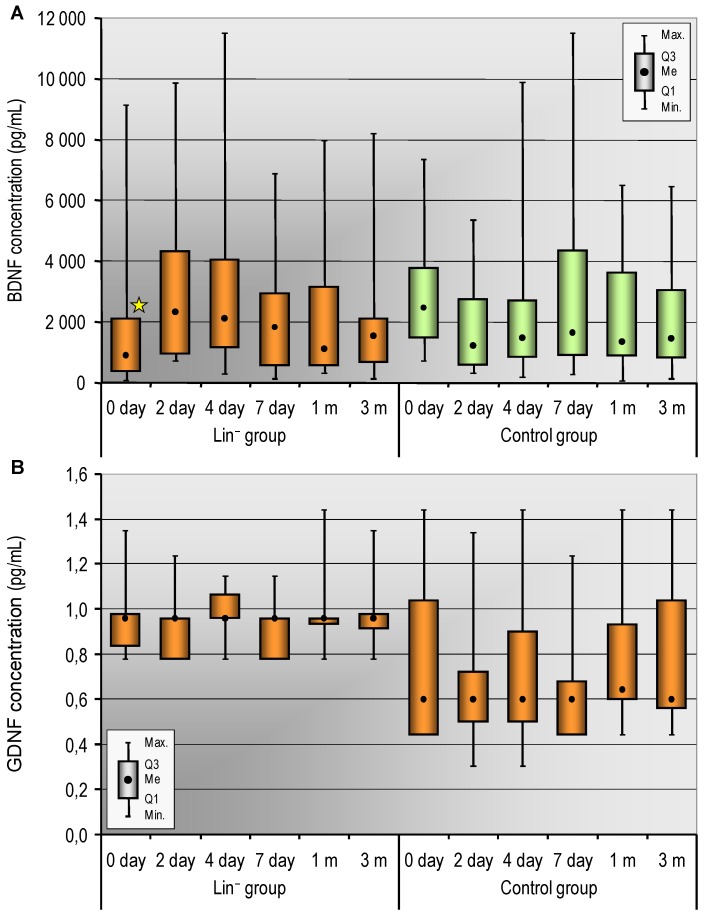
(**A**) The plasma levels of BDNF in the Lin^−^ group and in the control group (day 0, 2, 4, and 7 and in month 1 and 3); m: month. Data are presented as median (lower – upper quartile). * *p* < 0.05 for BDNF concentration in the Lin^–^ group vs. control group on day 0 (Mann–Whitney U-test). (**B**) The plasma levels of GDNF in Lin^−^ group and in the control group (day 0, 2, 4, and 7 and in month 1 and 3). Data are presented as medians (lower–upper quartiles).

In order to assess the influence of the intra-arterial administration of Lin^−^ cells in patients with STEMI on the plasma concentration of selected neurotrophins, we evaluated BDNF and GDNF levels on the day 0, 2, 4, and 7 as well as after 1 and 3 months. We found that the baseline concentration of BDNF on day 0 before using Lin^−^ cells was significantly higher in the control group than in the Lin^−^ study group. However, in the following days, we did not find any significant differences between the groups in the range of BDNF concentrations in subsequent time points (Table 4, Figure 1A). Next, we analyzed the dynamics of changes in BDNF concentrations in the control group vs. the Lin^−^ group. The level of BDNF in the control group tended to reduce on day 2 in relation to day 0 (borderline significance in Friedman ANOVA, *p* = 0.02 in Wilcoxon signed-rank test). In contrast, in the Lin^−^ group on day 4, we observed a tendency for the plasma level to be increased in BDNF in relation to day 0 (borderline significance in Friedman ANOVA, *p* = 0.01 in Wilcoxon signed-rank test) (Table 4 and Table 5).

In parallel, we analyzed the plasma concentrations of GDNF. The baseline concentration of GDNF on day 0 before using Lin^−^ cells was significantly higher in the study group than in the control group. The concentration of GDNF in following days remained at the same level in both groups (Table 4, Figure 1B).

Summing up, the plasma level of BDNF tended to be increased in the study group in the first days of therapy, contrary to the control group where a steady decrease of BDNF was noticed. There were no changes in plasma GDNF concentration after cell therapy.

### 2.4. Plasma Level Profiles of Angiogenic Growth Factors

Next, we analyzed the concentration of angiogenic growth factors in peripheral blood of patients with STEMI. We found that the baseline VEGF level was higher in the control group than in the Lin^−^ group (median 18.3 (15.6) pg/mL vs. 7.6 (7.6) pg/mL, *p* = 0.033) (Table 4). However, we did not observe differences between these groups in other time points. Similarly, baseline angiopoietin-1 levels were also significantly higher in the control group than in the Lin^−^ group, but in further observation we found that angiopoietin levels in the Lin^−^ group were higher than in the control group on day 2. As Tie-2 is concerned, we observed insignificantly higher levels of the factor in the control group on day 0, and then we did not observe any differences between the plasma levels in both groups. Interestingly, endoglin levels were higher in the control group than in the Lin^−^ group in every time point. There were no differences between endothelin levels between both groups of patients (Table 4).

We noticed a tendency for HGF levels to be higher in Lin^−^ patients in month 3 of observation compared to the control group (median 105 (58) pg/mL vs. 85.7 (46.5) pg/mL, *p* = 0.051); however, this difference was not statistically significant. Insulin-like growth factor binding-protein 1 (IGFBP-1) levels were higher in the Lin^−^ group than in control group at day 0 (*p* = 0.023).

**Figure 2:**
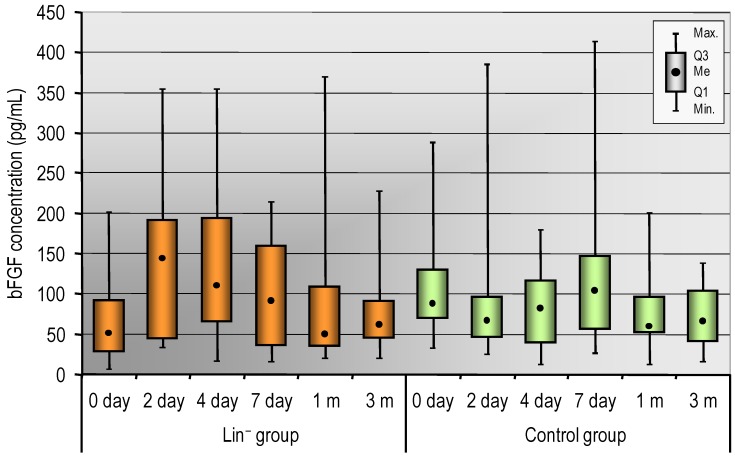
The plasma levels of bFGF in the Lin^−^ group and in the control group (day 0, 2, 4, and 7 and in month 1 and 3); m: month. Data are presented as medians (lower – upper quartiles).

Baseline basic fibroblast growth factor (bFGF) levels tended to be higher in the control group than in the Lin^−^ group (*p* = 0.051). In contrary, there was a tendency for bFGF plasma levels to be higher in the Lin^−^ group on day 4 (*p* = 0.056) (Table 4, Figure 2). Differences in bFGF concentrations (days 2 and 4 vs. day 0; and months 1 and 3 vs. day 0) were significantly different between the groups (*p* = 0.006, *p* ≤ 0.001; and *p* = 0.03, *p* = 0.036, respectively). Similarly, baseline platelet-derived growth factor-aa (PDGF-AA) levels were higher in the control group compared to the Lin^−^ group, and, subsequently, we observed the tendency for these levels to be higher in the Lin^−^ group.

Next, we analyzed changes in growth factor levels during the time of observation. We noticed a significant increase in plasma levels of VEGF, angiopoietin-1, bFGF, and platelet-derived growth factor-aa (PDGFA-AA) in Lin^−^ patients mostly from day 2 to 4 compared to baseline level (day 0) (Table 4).

### 2.5. Correlation between Selected Neutrotrophins and Angiogenic Factor Concentration with the Number of Administered Lin^−^ cells

Next, we focused on factors that could have influenced concentrations of neurotrophins and angiogenic factors. Correlation analysis showed that the increase in BDNF concentration observed in the Lin^−^ group one month after therapy compared to baseline concentration significantly correlated with the number of administered Lin^−^ cells (Spearman’s correlation coefficient, Rs = 0.55, *p* = 0.034). The more Lin^−^ cells that were infused, the higher the increase of BDNF concentration was at month 1 in relation to baseline.

Similarly, correlation analysis showed that the increase in bFGF plasma levels observed in the Lin^−^ group one month after therapy compared to baseline level significantly correlated with the number of administered Lin^−^ cells (Spearman’s correlation coefficient, Rs = 0.56, *p* = 0.029). The more Lin^−^ cells that were infused, the higher the increase of bFGF concentration was after one month in relation to baseline.

## 3. Discussion

Irreversible death of cardiomyocytes and scar formation due to prolonged ischemia of myocardium in consequence lead to adverse remodeling of the left ventricle. Contractile dysfunction of the left ventricle and cardiac arrhythmias represent common results of remodeling and most serious complications in patients with heart failure [27]. Strategies to prevent of remodeling, consecutive heart failure, and sudden death are urgently required. It has been expected that stem cell-based therapy will benefit patients by modifying adverse cardiac remodeling. Stem/progenitor cells used in therapeutic approaches could initiate immunomodulatory mechanisms, thereby reducing remodeling and having cardioprotective effects. One of mechanisms probably involved in the process is the secretion of growth and trophic factors including neurotrophins and angiogenic factors [14]. Therapeutic results associated with stem cell therapy, mainly due to paracrine mechanisms, might play an important role in the future [28].

The last two decades have brought an increased interest in neurotrophic factors. Neurotrophins (NTs) play an essential role as a regulator of cell survival and maintenance of their physiological activity. One of the most potent NTs is BDNF. Although the expression of BDNF primarily affects the central nervous system, this unique factor is also present in serum, which has been confirmed in numerous clinical and preclinical studies [29,30,31]. As potential sources of BDNF, blood platelets, brain neurons, and vascular endothelial cells are mentioned [32]. Fujimura observed that activated blood platelets may bind, store, and release BDNF [33]. However, the values of BDNF concentration in blood plasma depend on many factors, among others mental illness, eating disorders, lower respiratory tract infections, and physical activity [29,34,35,36]. Reduced plasma levels of neurotrophins, such as nerve growth factor (NGF) and BDNF, have been reported in patients with ACS [37]. These observations support the hypothesis that BDNF may be implicated in the pathogenesis of human coronary atherosclerosis. It has been demonstrated that BDNF stimulates formation of new vessels through an increase of VEGF concentration. Otherwise, the results reported by Kermani et al. have suggested that local regional delivery of BDNF may provide a novel mechanism for inducing neoangiogenesis through both direct actions on local TrkB-expressing endothelial cells in skeletal muscle and recruitment of specific subsets of TrkB^+^ BM-derived hematopoietic cells to provide peri-endothelial support for the newly formed vessels [38]. Moreover, BDNF concurs to increase factors that promote survival of cardiomyocytes and stimulate angiogenesis resulting in a decrease of remodeling of heart after myocardial infarction [39].

It is commonly speculated that benefits related to administration of SPCs are associated predominantly with their paracrine effects. Currently, it is widely believed that the paracrine effect—understood as the release of cytokines, chemokines, and growth factors inhibiting apoptosis and fibrosis, which enhances contractility and activates regenerative mechanisms—plays a pivotal role in stem cell-based therapy [40]. Secretion of cytoprotective factors by mesenchymal stem cells (MSCs) was first reported by Gnecchi et al. [41,42]. Released growth factors increase the survival of nearby cardiomyocytes, promote angiogenesis, and improve left ventricular function [43,44,45]. It has been proven that paracrine factors derived from stem cells transplanted into the myocardium contribute to left ventricular remodeling and function [45].

In this study, we assessed whether cellular therapy affected the levels of selected neurotrophins in patients undergoing application of autologous, BM-derived Lin^−^ SPCs into the coronary artery in the acute phase of myocardial infarction. We observed an increase in BDNF concentration on days 2 and 4 in patients that were administered Lin^−^ cells into the infarct-related artery (statistically insignificant). Whereas, the level of BDNF in the control group tended to be reduced on these days. This observation may suggest the short-acting trophic support derived from administered SPCs. Moreover, we also noticed that the increase in BDNF concentration one month after therapy significantly correlated with the number of administered Lin^−^ cells. The more Lin^−^ cells that were infused, the higher the increase of median BDNF concentration was at month 1 in relation to baseline. These observations support the hypothesis that the Lin^−^ cells used had short-acting as well as long-acting benefits. In previous studies, it has been suggested that BDNF protects the myocardium against ischemic injury [46,47]. BDNF expression is probably upregulated by neural signals from the heart after myocardial infarction. The BDNF/TrkB axis has been demonstrated to alleviate cardiac ischemic injury and inhibit cardiomyocyte apoptosis by regulating TRPC3/6 channels. BDNF has inhibited cardiomyocyte apoptosis by upregulating Bcl-2 expression and downregulating caspase-3 expression and activity in ischemic myocardium [47]. A complementary explanation for the cardioprotective activity of BDNF could be an interplay between BDNF and miR-195 in ischemic cardiomyocyte apoptosis. It has been shown that upregulation of miR-195 in ischemic cardiomyocytes promotes ischemic apoptosis by targeting Bcl-2, and BDNF mitigates the pro-apoptotic effect of miR-195 [48].

Another neurotrophin, a member of the transforming growth factor superfamily, GDNF, promotes the survival of developing sympathetic neurons and the growth of neurites from these neurons in culture. GDNF also acts as a potent chemoattractant for axon guidance of sympathetic neurons both in vitro and in vivo, and it exerts strong effects to navigate sympathetic axons to target cardiac tissues in the case of myocardial damage or transplantation of regenerated myocardial tissue [49]. Recently, it has been demonstrated that GDNF receptor GFRA2 is essential in heart development [50]. However, we did not find any increase in GDNF plasma concentration in patients that received Lin^−^ cells.

Growth factors are crucial in the development and regeneration of every tissue. Evidence has accumulated that various cytokines released by SPCs, such as growth factors, stimulate angiogenesis and protect the myocardium against ischemia. Data from animal models indicate that regenerative responses of endogenous cardiac stem cells (CSCs) can be enhanced by the administration of growth factors in situ. CSCs have been shown to possess receptors for IGF-1 and HGF, factors that regulate their growth, survival, and migration [23]. Activation of signaling pathways deriving from their receptors was confirmed by phosphorylation of c-met protein and further transmitters. There is accumulating evidence that such a regenerative response can be activated in myocardial ischemia [24,51]. The mitogenic potential to stimulate HGF proliferation and the cytoprotective effects of IGF-1 appear to be a complementary combination necessary in cardiac repair processes.

Inducing angiogenesis is one of the biggest challenges for modern therapy. It seems that only use of stem cells and growth factors can compete in this challenge. Recently, great therapeutic potential has been attributed to VEGF [52]. Data from preclinical and clinical studies indicate that VEGF may induce angiogenesis in myocardial infarction [52]. Besides its prominent role in angiogenesis, VEGF also exerts important mobilizing effect on stem cells. It is one of the factors that mobilizes BM stem cells and participates in the recruitment of these cells to sites of damaged tissue in STEMI. Angiopoietin-1 is another factor that stimulates angiogenesis and promotes the maturation of blood vessels [19,22]. A unique, advantageous feature of angiopoietin-1 is stabilization of blood vessels and protection from VEGF-induced plasma leakage [53]. Angiogenic and anti-apoptotic properties of HGF have been demonstrated [54]. Previous studies have shown a gradual increase in the endogenous concentration of HGF mRNA and its receptors (c-met) for up to one week after myocardial infarct [55]. It has also been shown that the expression of HGF and its secretion into the blood circulation are promoted in the early phase of AMI [55].

bFGF is an endogenous, multifunctional protein with strong affinity for the extracellular matrix and basal lamina as well as well-documented paracrine, autocrine, and intracellular modes of action [56]. It has been demonstrated that bFGF exerts acute and direct prosurvival effects in ischemic myocardium. bFGF has also been shown to be a potent angiogenic protein and a crucial agent for the proliferation, expansion, and survival of several cell types including those with stem cell properties [57]. It has also been demonstrated that bFGF has cytoprotective and regenerative properties [55]. In the study, we noticed that the increase in bFGF concentration one month after SPC intracoronary application significantly correlated with the number of administered Lin^−^ cells. The more Lin^−^ cells that were infused, the higher the increase of BDNF concentration was at month 1 in relation to baseline. These observations support the hypothesis that autologous, BM-derived Lin^−^ may be a source of angiogenic and trophic factors for damaged myocardium. Given the cytoprotective and regenerative properties of bFGF, intracoronary administration of SPCs may exert beneficial effects on the myocardium.

## 4. Materials and Methods

### 4.1. Patients

The study was designed as a prospective, open-label, nonrandomized clinical trial in a single center for subjects with STEMI. The trial was approved by the Ethics Committee of the Pomeranian Medical University in Szczecin (Poland) and performed in accordance with the Declaration of Helsinki (BN-001/122/05, 20.06.2005). All patients provided written, informed consent.

A total of 34 patients, all males without diabetes, between 35 and 63 years old (mean 52 ± 7.7) with STEMI were enrolled in the study. The survey was carried out from December 2010 to June 2014 at the Department of Cardiology at Pomeranian Medical University in Szczecin. The participants were divided into two groups: a study group (standard cardiovascular treatment with autologous Lin^−^ stem cell transplantation—15 patients) and a control group (standard cardiovascular treatment solely—19 patients). Patients enrolled in the study met the following criteria:

(a) under 65 years of age;
(b) occurrence of a typical angina pectoris lasting at least 30 min and the appearance of chest pain up to 12 h before admission to the clinic;
(c) elevation of the ST segment at point J > 0.2 mV in at least two adjacent leads from V1 to V3 or >0.1 mV in other electrocardiogram (ECG) leads;
(d) first ever myocardial infarction;
(e) ejection fraction (EF) ≤45% on day 0 of echocardiographic examination; and
(f) single-vessel coronary disease in coronary angiography qualified for coronary angioplasty with stent implantation.

On the day of admission, clinical conditions of all enrolled patients were assessed according to Killip Kimball’s classification and heart ultrasound examination. Subsequently, patients underwent coronary angiography and percutaneous revascularization with the implantation of drug eluting stent (DES) to the infarcted artery. Coronarography was performed using the Judkins technique on Integris HM hardware (Philips Healthcare, Amsterdam, Netherlands). The recording from the study was stored at a speed of 25 frames/s. Angiograms were analyzed offline using DICOM 3 software (Medical Imaging & Technology Alliance, Arlington, VA, USA). The flow through the infarct-related coronary artery was evaluated in the initial angiogram and after the primary angioplasty based on the Thrombosis In Myocardial Infarction (TIMI) scale. Primary angioplasty was performed according to generally accepted principles in patients with TIMI 0 flow (fully obstructed coronary artery). In each patient, during the procedure, only the infarct-related artery was supplied. In the study group, 10 patients had a left anterior descending coronary artery (LAD), 2 patients had a left circumflex artery (LCX), and 3 patients had a right coronary artery (RCA) supplied. In the control group, 14 patients had LAD, 1 patient had LCX, and 4 patients had RCA supplied.

### 4.2. Cells

Bone marrow from the 15 patients in the study group was obtained within 24 h after coronary angioplasty. Each time, informed consent was given. BM samples (40–50 mL) were aspirated in local anesthesia from the posterior iliac crest of recruited patients and subsequently resuspended in a collecting medium (phosphate-buffered saline (PBS), pH 7.2) and heparin (20 U/mL; Gibco, Waltham, MA, USA). MNCs were obtained after centrifugation over Gradisol L (Polfa, Kutno, Poland) [58]. The obtained suspension of BM MNCs was subjected to immunomagnetic separation procedures (MiniMACS, Miltenyi Biotec, Auburn, AL, USA). Lin^−^ cells were isolated from MNCs using immunomagnetic isolation and a lineage cell depletion kit (Miltenyi Biotec, Auburn, AL, USA), as described [15], according to good medical practice (GMP) conditions. Before implantation, the cells were maintained in 2 mL of sterile PBS. According to the experimental protocol, all isolated Lin^−^ cells were administered into the infarct-related coronary artery each time, which had been previously subjected to coronary angioplasty. Table 2 shows the phenotypic characterization of administered Lin^−^ cells. Lin^−^ cells are a very heterogeneous population that is highly enriched for immature SPCs. In our previous work, by employing flow cytometry, we have shown that Lin^−^ cells contain populations of CD34^+^ cells (12.1% ± 7.2%), CD133^+^ cells (12.3% ± 8.2%), endothelial progenitor cells (CD34^+^, CD133^+^, and CD144^+^ cells, 1.7% ± 1.1%), and cells with mesenchymal stem cell phenotypes (CD105^+^, CD73^+^, CD90^+^, CD45^−^, CD34^−^, CD11b^−^, CD19^−^, and HLA-DR^−^ cells; 0.0084% ± 0.0108%) [15].

### 4.3. Administration of the Lin^−^ Stem Cells Suspensio to the Infarct-Related Artery

Administration of isolated Lin^−^ SPCs to the patient was performed up to 24 h after coronary angioplasty. Lin^−^ cells were infused into the infarct-related coronary artery, which had been previously subjected to coronary angioplasty. A typical set for coronary angioplasty, with a guide catheter and an over-the-wire (OTW) balloon dilatation catheter (Boston Scientific Emerge TM), was used to implant stem cells. After placing the balloon of the OTW catheter within the previously implanted stent, it was expanded. The prepared suspension of Lin^−^ cells was administered in a sterile fashion through the catheter flow channel distal to the balloon. Deflation of the balloon was performed 2 min after the suspension was administered, restoring blood flow in the artery. The control group was not subjected to another coronary angiography within 24 h to stop the flow in the artery solely due to ethical reasons.

### 4.4. Ultrasound Assessment of the Heart

In all patients, echocardiography was performed on the day of admission to the Department of Cardiology (day 0—initial examination). Subsequent echocardiographic examinations were carried out in all patients on days 1, 3, and 7 as well as after 1, 3, 6, and 12 months after myocardial infarction. Echocardiographic examination was performed each time by the same professional that was blinded to the treatment arm using an Acouson 128 XP/10c apparatus with cardiac transducer 2/5/3.5 MHz. The study was performed in standard projections: apical four-chamber, dual-chamber, and long and short axes in two-dimensional mode. Results were recorded on a CD and analyzed both online and offline. The left ventricular function was assessed based on the left ventricular ejection fraction (LVEF), end-diastolic volume (LVEDV), end-systolic volume (LVESV), and diameter of the aortic bulb (Ao) obtained with Simpson’s two-dimensional method in four-chamber and two-chamber apical projections using Acouson software.

### 4.5. Holter ECG Examination

In addition, all patients included in the study in the first day and after 6 and 12 months underwent a 24 h Holter ECG. Oxford apparatus and software were used for the study (Oxford Pol Sp. z o.o., Poland).

### 4.6. Assessment of Plasma Concentration of Selected Neurotrophins, Growth, and Chemotactic Factors Using Luminex

Angiopoietin-1, bFGF, platelet-derived growth factor-aa (PDGF-AA), and VEGF concentrations were quantified by multiplex fluorescent bead-based immunoassays (Luminex Corporation, Austin, TX, USA) using commercial Human Angiogenesis Premixed Kit A Magnetic Luminex Performance Assay (R&D Systems, Minneapolis, MN, USA) at various time points (day 0, 2, 4, and 7; month 1 and 3). Human Premixed Multi-Analyte Kit Magnetic Luminex Assay (R&D Systems, Minneapolis, MN, USA) was used to detect the concentrations of BDNF, endoglin, endothelin-1, GDNF, HGF, insulin-like growth factor binding-protein 1 (IGFBP-1), and Tie-2 using the Luminex system. The procedures were performed according to the manufacturer’s protocol.

### 4.7. Statistics

Chi-square or Fisher’s exact tests were used to compare qualitative variables. Since the number of patients in each group was too low to reliably assess the normality of distributions of quantitative variables, non-parametric tests were used, and data were presented as median (interquartile range—IQR). Mann–Whitney tests were used to compare quantitative variables between groups. Differences of parameters measured on day 0 and on subsequent days of observation in each patient (delta values) were calculated and compared between groups to study the dynamics of changes. Significance of the differences within each group was assessed with repeated-measures Friedman ANOVA, which was followed, in the case of significant (*p* < 0.05) differences between time points, by Wilcoxon signed-rank tests for comparison to baseline (day 0) values. Spearman’s rank correlation coefficient (Rs) was used to measure the strength of associations between quantitative variables within groups. A *p* < 0.05 was considered statistically significant.

## 5. Limitations

Overall, our study gave some interesting results, but this was not without some drawbacks. The first is the diversity among the investigated groups. Patients enrolled in the study differed considerably in terms of age (from 35 to 63 years old) and coronary artery supplied. Relatively low numbers of injected Lin^−^ stem/progenitor cells varied in each particular patient. Additionally, it should be mentioned that the application of Lin^–^ cells containing a significant percentage of progenitor cells, although relatively easy to obtain, does not fully reflect the results obtained using a pure fraction of stem cells. Due to lack of randomization, the analysis showed the differences in baseline patient characteristics, which significantly impeded analysis of obtained results. The double-blinded, placebo-controlled, randomized study might have reduced this limitation to some extent. Another factor that might have affected the results is the limited number of recruited patients. Overall, this study was not designed, nor was it large enough, to determine the efficacy of Lin^−^ stem/progenitor cells on myocardial regeneration or LVEF improvement. Additional studies are warranted to address this study limitation. The final drawback was the lack of subsequent angiography within 24 h in the control group due to ethical reasons.

## 6. Conclusions

Altogether, we have not observed any adverse effects of autologous, BM-derived Lin^−^ stem/progenitor cells delivered intracoronarily, which makes them a safe and feasible source for cell therapy. There were no deaths, severe adverse effects, transplant-related infections, acute kidney injuries, and subsequent AMI during one-year follow up. No evidence of ventricular or supraventricular arrhythmias in 24 h ECG monitoring in the first day and after 6 and 12 months were noted.

We found a tendency for BDNF plasma levels to be increased in patients treated with Lin^−^ stem/progenitor cells in the initial days of the infarction, while in the control group we observed a trend for BDNF levels to be reduced. In parallel, we noticed significant increases in plasma levels of VEGF, angiopoietin-1, bFGF, and PDGF-AA in Lin^−^ patients mostly from day 2 to 7. However, we did not find any increase in GDNF plasma concentration in patients that received Lin^−^ stem/progenitor cells. The increase in the plasma concentrations of neurotrophic, angiopoetic, and anti-apoptotic factors in most cases took place until day 7 of the experiment. This relatively short period of increased paracrine function of progenitor cells may prove to be crucial for patients with AMI. Likewise, the fastest possible revascularization increases the chances of survival and full recovery of patients.

In addition, we confirmed that in the study group the increases in BDNF and bFGF levels in the first month of observation significantly depended on the number of autologous Lin^−^ cells that were administered. The obtained number of Lin^−^ stem/progenitor cells from the same BM volume differs among patients for individual reasons, linearly decreases with age, and depends on coexisting diseases and their severity. In future studies, aspiration of larger amounts of BM should be considered to obtain higher amounts of Lin^−^ stem/progenitor cells.

To sum up the clinical effects of this experiment, we showed that at 6 and 12 months of observation the heart ventricles in the control group began to undergo unfavorable reconstruction by increasing their diameter. Similarly, the diameter of the aortic bulb tended to be increased in the control group at six months. This might be the result of the beneficial, long-term effect of increased levels of several angiogenic factors in the study group. Higher concentrations of trophic factors in plasma may protect the myocardium against ischemic injury [46,47], indirectly promote survival of cardiomyocytes, and stimulate angiogenesis, resulting in a decrease of remodeling of the heart after myocardial infarction [39].

Taken together, these findings suggest that the administered SPCs may be a valuable source of angiogenic and trophic factors for damaged myocardium, although this observation requires further in-depth studies.

## Figures and Tables

**Table 1:** Clinical characteristics of the study groups.

Characteristics	Lin^−^ Group (*n* = 15)	Control Group (*n* = 19)	*p* Value
Age (y) median (IQR)	54.5 (11.3)	52 (11.5)	0.78
**Cardiovascular Risk Factors**			
Hypertension *n* (%)	5 (33.3)	6 (31.6)	0.91
Smoking *n* (%)	8 (53.3)	10 (52.6)	0.97
Diabetes mellitus *n* (%)	0 (0.0)	0 (0.0)	—
Family history of coronary artery disease (CAD) *n* (%)	7 (46.7)	10 (52.6)	0.73
**Quantitative parameters**	**Median (IQR)**	**Median (IQR)**	***p* value**
Creatine kinase muscle-brain (CKMB) (U/L)	173.5 (245.3)	379.0 (312.5)	0.15
Troponin I (TN-I) (µg/L)	9.9 (16.3)	19.9 (10.3)	0.23
Brain natriuretic peptide (BNP) (pg/mL)	1210.0 (1437.3)	1602.5 (1247.3)	0.78
Lipid profile:			
Total cholesterol (mg/dL)	236.0 (61.5)	211.5 (79.0)	0.10
Low-density lipoprotein (LDL) cholesterol (mg/dL)	142.5 (60.5)	140.0 (65.8)	0.56
High-density lipoprotein (HDL) cholesterol (mg/dL)	46.5 (15.3)	40.0 (9.8)	0.17
Triglycerides (mg/dL)	149.0 (90.0)	117.5 (87.0)	0.02
Left ventricular end-systolic volume (LVESV) (mL)	74.0 (22.3)	90.5 (16.0)	0.04
Ejection fraction (EF) (%)	40.0 (5.8)	35.0 (3.8)	0.05
Left ventricular end-diastolic volume (LVEDV) (mL)	128.5 (39.3)	151.5 (31.8)	0.03
**Qualitative parameters**	***n* (%)**	***n* (%)**	***p* value**
Infarction site:			
Anterior *n* (%)	10 (66.7)	14 (73.7)	0.20
Inferior *n* (%)	4 (26.7)	4 (21.1)	
Lateral *n* (%)	1 (6.7)	1 (5.3)	
Coronary artery:			
Left anterior descending (LAD) *n* (%)	10 (66.7)	14 (73.7)	0.71
Right coronary artery (RCA) *n* (%)	3 (20.0)	4 (21.1)	
Left circumflex artery (LCX) *n* (%)	2 (13.3)	1 (5.3)	

Mann–Whitney U-test for quantitative variables or Fisher exact test for qualitative variables; *p* value—Lin^−^ group vs. control group.

**Table 2:** The phenotypic characterization of administered autologous Lin^−^ cells.

Cell Population	Phenotypic Characterization	Function
**Lin^+^ cells**	CD2^+^, CD3^+^, CD11b^+^, CD14^+^, CD15^+^, CD16^+^, CD19^+^, CD56^+^, CD123^+^, CD235a^+^ (Glycophorin A)	Mature hematopoietic cells such as T cells, B cells, NK cells, dendritic cells, monocytes, granulocytes, erythroid cells, and their committed precursors.
**Lin^–^ cells**	CD2^–^, CD3^–^, CD11b^–^, CD14^–^, CD15^–^, CD16^–^, CD19^–^, CD56^–^, CD123^–^, CD235a^–^ (Glycophorin A)	The fraction does not contain any morphotic elements exhibiting mature phenotypes. The depletion of lineage-positive cells results in the enrichment of precursor cells, progenitor cells, and stem cells such as CD34^+^ and CD133^+^ cells.

**Table 3:** The selected ultrasonographic parameters in the Lin^−^ group and in the control group (day 0, 1, 3, and 7 and in month 1, 3, 6, and 12).

Groups	0 Day	1st Day	3rd Day	7th Day	1st Month	3rd Month	6th Month	12th Month
**EF (%)**								
Lin^−^ group	40.0 (5,8)	40.0 * (6.0)	41.0 * (9.0)	44.0 * (10.5)	46.0 * (10.8)	49.0 * (14.5)	49.0 * (15.8)	49.5 * (18.0)
Control group	35.0 (3.8)	39.5 * (5.0)	40.5 * (4.3)	43.0 * (6.3)	42.0 * (8.0)	44.5 * (10.0)	45.0 * (14.0)	46.0 * (15.5)
*p* value	0.047	0.147	0.560	0.471	0.410	0.430	0.584	1.000
**LVEDV (mL)**								
Lin^−^ group	128.5 (39.3)	128.0 (36.8)	129.0 (35.0)	122.5 (34.3)	125.5 (25.3)	113.5 (38.3)	119.5 (47.5)	114.5 (42.5)
Control group	151.5 (31.8)	146.5 * (41.8)	136.5 * (41.8)	131.0 * (44.3)	130.5 * (36.8)	129.0 * (43.3)	123.5 * (37.5)	124.0 * (39.3)
*p* value	0.025	0.071	0.286	0.215	0.242	0.372	0.758	0.732
**LVESV (mL)**								
Lin^−^ group	74.0 (22.3)	72.0 * (10.3)	70.0 (26.5)	64.5 * (21.5)	62.0 * (21.3)	55.5 * (37.3)	48.5 * (41.5)	49.5 (41.0)
Control group	90.5 (16.0)	85.0 * (20.5)	79.5 * (20.3)	73.5 * (17.5)	72.5 * (22.5)	70.5 *(25.3)	66.0 * (25.5)	64.5 * (30.0)
*p* value	0.043	0.025	0.157	0.157	0.256	0.256	0.632	0.681
**LVIDD (mm)**								
Lin^−^ group	51.0 (4.0)	51.0 (6.8)	52.5 (7.3)	54.0 (8.3)	55.0 (7.3)	52.5 (8.8)	49.5 (9.8)	53.0 (9.5)
Control group	56.0 (4.0)	55.0 (6.0)	53.5 * (7.0)	54.5 * (7.3)	54.5 (9.8)	58.0 (7.3)	55.5 (10.3)	58.0 (9.0)
*p* value	0.007	0.047	0.302	0.864	0.784	0.167	0.051	0.051
**Diameter of the aortic bulb (mm)**								
Lin^−^ group	34.0 (3.3)	33.0 (4.8)	34.0 (7.3)	35.0 (5.3)	34.5 (6.0)	34.0 (5.3)	35.5 (5.0)	33.0 (5.8)
Control group	36.5 (6.0)	36.5 (6.0)	35.0 (5.8)	36.0 (5.8)	36.0 (3.8)	36.0 (5.3)	37.0 (5.5)	35.0 (5.0)
*p* value	0.157	0.043	0.354	0.372	0.410	0.111	0.066	0.071

Data are expressed as median (IQR); p value—Lin^−^ group vs. control group, Mann–Whitney U-test; * *p* < 0.05 for difference between 0 day and subsequent time points using Friedman ANOVA followed by Wilcoxon signed-rank tests; for all differences significant in the Wilcoxon signed-rank test, Friedman ANOVA also yielded *p* < 0.05.

**Table 4:** The plasma concentrations of brain-derived neurotrophic factor (BDNF), glial-derived neurotrophic factor (GDNF), vascular endothelial growth factor (VEGF), angiopoietin-1, hepatocyte growth factor (HGF), insulin-like growth factor binding-protein 1 (IGFBP-1), endoglin, platelet-derived growth factor-aa (PDGF-AA), angiopoietin-1 receptor (Tie-2), endothelin-1, and bFGF in the Lin^−^ group and in the control group (day 0, 2, 4, and 7 and in month 1 and 3).

Groups	0 Day	2nd Day	4th Day	7th Day	1st Month	3rd Month	Friedmann ANOVA *p* Value
**BDNF (pg/mL)**							
Lin^−^ group	882 (1774)	2319 (3400)	2094 (2898)	1809 (2402)	1099 (2621)	1530 (1462)	0.075
Control group	2444 (2312)	1201 (2188)	1464 (1871)	1614 (3461)	1331 (2785)	1427 (2259)	0.088
*p* value	0.019	0.120	0.336	0.811	0.656	0.732	
**GDNF (pg/mL)**							
Lin^−^ group	0.96 (0.14)	0.96 (0.18)	0.96 (0.11)	0.96 (0.18)	0.96 (0.02)	0.96 (0.07)	0.63
Control group	0.60 (0.60)	0.60 (0.22)	0.60 (0.40)	0.60 (0.24)	0.64 (0.33)	0.60 (0.48)	0.28
*p* value	0.030	0.003	0.005	0.001	0.015	0.025	
**VEGF (pg/mL)**							
Lin^−^ group	7.6 (7.6)	17.2 (18.8)	20.3 * (23.4)	15.7 (20.8)	9.4 (10.2)	10.6 (14.6)	0.01
Control group	18.3 (15.6)	9.8 (10.3)	15.0 (19.1)	17.6 (37.3)	9.2 * (11.6)	9.0 (14.1)	0.001
*p* value	0.033	0.120	0.354	0.372	0.837	1.000	
**Angiopoietin-1 (pg/mL)**							
Lin^−^ group	1159 (1211)	3714 (3135)	3165 * (3999)	2539 (4839)	1122 (2958)	1502 (1637)	0.03
Control group	2868 (2529)	1965 * (1454)	2450 (3172)	3799 (5003)	1437 * (2314)	1783 (2022)	0.005
*p* value	0.012	0.060	0.147	0.537	1.000	1.000	
**HGF (pg/mL)**							
Lin^−^ group	182 (178)	170 * (136)	141 * (61)	130 * (103)	112 * (40)	105 * (58)	0.00001
Control group	310 (556)	214 * (250)	151 * (136)	124 * (108)	93 * (59)	86 * (46)	0.00001
*p* value	0.168	0.560	0.918	0.472	0.515	0.051	
**IGFBP-1 (pg/mL)**							
Lin^−^ group	29542 (15747)	8945 * (20426)	4146 * (13070)	20443 (17910)	21248 (25772)	21570 (22494)	0.001
Control group	13817 (20845)	13062 (15269)	11479 (17643)	13507 (12931)	10491 (8228)	11120 (10802)	0.54
*p* value	0.023	1.000	0.354	0.215	0.256	0.147	
**Endoglin (pg/mL)**							
Lin^−^ group	903 (702)	849 * (707)	835 * (590)	965 (814)	842 (528)	1018 (589)	0.0003
Control group	1256 (553)	1191 (565)	1108 * (366)	1185 * (295)	1176 (394)	1282 (427)	0.03
*p* value	0.056	0.056	0.025	0.103	0.010	0.040	
**PDGF-AA (pg/mL)**							
Lin^−^ group	119 (162)	300 * (439)	291 * (361)	266 (383)	116 (325)	138 (179)	0.02
Control group	248 (277)	165 (125)	211 (206)	296 (344)	166 (252)	150 (227)	0.11
*p* value	0.033	0.096	0.104	0.810	0.706	0.946	
**Tie-2 (pg/mL)**							
Lin^−^ group	283 (289)	416 (969)	440 (725)	413 (290)	307 (218)	290 (324)	0.10
Control group	467 (472)	387 (512)	400 (207)	495 (906)	296 (415)	318 (323)	0.05
*p* value	0.096	0.918	0.864	0.190	0.758	0.354	
**Endothelin1 (pg/mL)**							
Lin^−^ group	5.3 (8.4)	4.7 (7.5)	5.9 (7.2)	5.9 (8.3)	5.3 (8.5)	6.6 (6.9)	0.30
Control group	6.8 (3.9)	5.9 (4.0)	5.9 (4.9)	6.3 (6.8)	6.8 (4.4)	6.8 (4.1)	0.19
*p* value	0.784	0.973	0.707	0.707	0.918	0.784	
**bFGF (pg/mL)**							
Lin^−^ group	51 (64)	144 * (147)	110 * (130)	90 (125)	50 (74)	61 (46)	0.04
Control group	88 (61)	70 * (51)	81 * (78)	104 (92)	76 * (46)	65 * (63)	0.004
*p* value	0.051	0.157	0.056	0.784	0.656	0.973	

Data are expressed as median (IQR); p value—Lin^−^ group vs. control group; Mann–Whitney U-test; Friedman ANOVA for differences between all time points was followed by Wilcoxon signed-rank test for difference between day 0 and subsequent time points (* *p* < 0.05).

**Table 5:** The differences between the following time points and day 0 (baseline) in plasma levels of BDNF, GDNF, VEGF, angiopoietin-1, HGF, IGFBP-1, endoglin, PDGF-AA, Tie-2, endothelin-1, and bFGF in the Lin^−^ group and in the control group.

The Differences between Following Time Points and Day 0 (Baseline)					
Groups	2nd Day	4th Day	7th Day	1st Month	3rd Month
**BDNF (pg/mL)**					
Lin^−^ group	1506	940	267	325	221
Control group	−987	−824	−795	−1167	−1157
*p* value	0.011	0.004	0.391	0.111	0.077
**GDNF (pg/mL)**					
Lin^−^ group	0	0	0	0	0
Control group	0	0	0	0	0
*p* value	0.945	0.336	0.706	0.758	0.732
**VEGF (pg/mL)**					
Lin^−^ group	4.91	6.02 *	3.08	−0.05	−0.23
Control group	−7.84	−0.72	−1.22	−7.04 *	−4.69
*p* value	0.019	0.025	0.837	0.066	0.111
**Angiopietin-1 (pg/mL)**					
Lin^−^ group	1978	1540 *	655	495	402
Control group	−853 *	−238	4.5	−887 *	−1419
*p* value	0.010	0.003	0.515	0.066	0.043
**HGF (pg/mL)**					
Lin^−^ group	−25.5	−46 *	−72 *	−64.1 *	−81.6 *
Control group	−94.5	−178.5 *	−219.5 *	−251.5 *	−271 *
*p* value	0.302	0.036	0.071	0.077	0.036
**IGFBP-1 (pg/mL)**					
Lin^−^ group	−16545 *	−20303 *	−7312	−8166	−11815
Control group	−1625	−2421	947	−1481	−1823
*p* value	0.027	0.030	0.319	0.319	0.515
**Endoglin (pg/mL)**					
Lin^−^ group	−99.5 *	−165 *	−80	−92.5	−58.5
Control group	−140.5	−198.5 *	−156 *	−92.5	−91
*p* value	0.973	0.837	0.515	0.784	0.493
**PDGF-AA (pg/mL)**					
Lin^−^ group	179.5 *	149.7 *	56.8	50.2	43.5
Control group	−56.3	6.5	14.5	−68.9	−133.1
*p* value	0.025	0.006	0.319	0.286	0.036
**Tie-2 (pg/mL)**					
Lin^−^ group	136.5	141	2.9	19	−9.9
Control group	−23	−52.5	61.5	−146.5	−157
*p* value	0.179	0.036	0.493	0.319	0.286
**Endothelin1 (pg/mL)**					
Lin^−^ group	0	0	0	0	0.83
Control group	−0.96	−0.24	−0.51	0	−0.72
*p* value	0.256	0.537	0.271	0.451	0.120
**bFGF (pg/mL)**					
Lin^−^ group	55.3 *	53.3 *	14.9	12.5	16.8
Control group	−24.6 *	−12.4 *	−3	−29.5 *	−43.6 *
*p* value	0.006	≤0.001	0.372	0.030	0.036

Data are expressed as medians; *p* value—Lin^−^ group vs. control group; Mann–Whitney U-test; * *p* < 0.05 for difference between day 0 and subsequent time points, Friedman ANOVA followed by Wilcoxon signed-rank test.

## Data Availability

The data used to support the findings of this study are available from the corresponding author upon request.

## References

[1] Jernberg T. (2016). Swedeheart Annual Report 2015.

[2] Pedersenn F., Butrymowicz V., Kebarek H., Wachtell K., Helqvist S., Kastrup I., Holmvany L., Clemmensen P., Engstrom T., Grande P. (2014). Short-and long term cause of death in patients treated with primary PCI for STEMI. J. Am. Coll. Cardiol..

[3] Sürder D., Manka R., Moccetti T., Lo Cicero V., Emmert M.Y., Klersy C., Soncin S., Turchetto L., Radrizzani M., Zuber M. (2016). Effect of Bone Marrow-Derived Mononuclear Cell Treatment, Early or Late After Acute Myocardial Infarction: Twelve Months CMR and Long-Term Clinical Results. Circ. Res..

[4] Xu J.Y., Liu D., Zhong Y., Huang R.C. (2017). Effects of timing on intracoronary autologous bone marrow-derived cell transplantation in acute myocardial infarction: A meta-analysis of randomized controlled trials. Stem Cell Res. Ther..

[5] Wojakowski W., Jadczyk T., Michalewska-Włudarczyk A., Parma Z., Markiewicz M., Rychlik W., Kostkiewicz M., Gruszczyńska K., Błach A., Dzier Zak-Mietła M. (2017). Effects of Transendocardial Delivery of Bone Marrow-Derived CD133+ Cells on Left Ventricle Perfusion and Function in Patients with Refractory Angina: Final Results of Randomized, Double-Blinded, Placebo-Controlled REGENT-VSEL Trial. Circ. Res..

[6] Tang J.N., Cores J., Huang K., Cui X.L., Luo L., Zhang J.Y., Li T.S., Qian L., Cheng K. (2018). Concise Review: Is Cardiac Cell Therapy Dead? Embarrassing Trial Outcomes and New Directions for the Future. Stem Cells Transl. Med..

[7] Sürder D., Manka R., Lo Cicero V., Moccetti T., Rufibach K., Soncin S., Turchetto L., Radrizzani M., Astori G., Schwitter J. (2013). Intracoronary injection of bone marrow-derived mononuclear cells early or late after acute myocardial infarction: Effects on global left ventricular function. Circulation.

[8] Jeyaraman M.M., Rabbani R., Copstein L., Sulaiman W., Farshidfar F., Kashani H.H., Qadar S.M.Z., Guan Q., Skidmore B., Kardami E. (2017). Autologous Bone Marrow Stem Cell Therapy in Patients With ST-Elevation Myocardial Infarction: A Systematic Review and Meta-analysis. Can. J. Cardiol..

[9] Wollert K.C., Meyer G.P., Müller-Ehmsen J., Tschöpe C., Bonarjee V., Larsen A.I., May A.E., Empen K., Chorianopoulos E., Tebbe U. (2017). Intracoronary autologous bone marrow cell transfer after myocardial infarction: The BOOST-2 randomised placebo-controlled clinical trial. Eur. Heart J..

[10] San Roman J.A., Sánchez P.L., Villa A., Sanz-Ruiz R., Fernandez-Santos M.E., Gimeno F., Ramos B., Arnold R., Serrador A., Gutiérrez H. (2015). Comparison of Different Bone Marrow-Derived Stem Cell Approaches in Reperfused STEMI. A Multicenter, Prospective, Randomized, Open-Labeled TECAM Trial. J. Am. Coll. Cardiol..

[11] Poole J.C., Quyyumi A.A. (2013). Progenitor Cell Therapy to Treat Acute Myocardial Infarction: The Promise of High-Dose Autologous CD34(+) Bone Marrow Mononuclear Cells. Stem Cells Int..

[12] Coskun V., Lombardo D.M. (2016). Studying the pathophysiologic connection between cardiovascular and nervous systems using stem cells. J. Neurosci. Res..

[13] Sanganalmath S.K., Bolli R. (2013). Cell therapy for heart failure: A comprehensive overview of experimental and clinical studies, current challenges, and future directions. Circ. Res..

[14] Gnecchi M., Zhang Z., Ni A. (2008). Paracrine mechanisms in adult stem cell signaling and therapy. Circ. Res..

[15] Paczkowska E., Kaczyńska K., Pius-Sadowska E., Rogińska D., Kawa M., Ustianowski P., Safranow K., Celewicz Z., Machaliński B. (2013). Humoral activity of cord blood-derived stem/progenitor cells: Implications for stem cell-based adjuvant therapy of neurodegenerative disorders. PLoS ONE.

[16] Majka M., Janowska-Wieczorek A., Ratajczak J., Ehrenman K., Pietrzkowski Z., Kowalska M.A., Gewirtz A.M., Emerson S.G., Ratajczak M.Z. (2001). Numerous growth factors, cytokines, and chemokines are secreted by human CD34(+) cells, myeloblasts, erythroblasts, and megakaryoblasts and regulate normal hematopoiesis in an autocrine/paracrine manner. Blood.

[17] Nagaya N., Kangawa K., Itoh T., Iwase T., Murakami S., Miyahara Y., Fujii T., Uematsu M., Ohgushi H., Yamagishi M. (2005). Transplantation of mesenchymal stem cells improves cardiac function in a rat model of dilated cardiomyopathy. Circulation.

[18] Urbich C., Aicher A., Heeschen C., Dernbach E., Hofmann W.K., Zeiher A.M., Dimmeler S. (2005). Soluble factors released by endothelial progenitor cells promote migration of endothelial cells and cardiac resident progenitor cells. J. Mol. Cell Cardiol..

[19] Zhou B.O., Ding L., Morrison S.J. (2015). Hematopoietic stem and progenitor cells regulate the regeneration of their niche by secreting Angiopoietin-1. Elife.

[20] Paczkowska E., Piecyk K., Łuczkowska K., Kotowski M., Rogińska D., Pius-Sadowska E., Oronowicz K., Ostrowski M., Machaliński B. (2016). Expression of neurotrophins and their receptors in human CD34+ bone marrow cells. J. Physiol. Pharmacol..

[21] Xie Y., Ibrahim A., Cheng K., Wu Z., Liang W., Malliaras K., Sun B., Liu W., Shen D., Cheol Cho H. (2014). Importance of cell-cell contact in the therapeutic benefits of cardiosphere-derived cells. Stem Cells.

[22] Brindle N.P.J., Saharinen P., Alitalo K. (2006). Signalling and functions of angiopoietin-1 in vascular protection. Circ. Res..

[23] Ellison G.M., Torella D., Dellegrottaglie S., Perez-Martinez C., Perez de Prado A., Vicinanza C., Purushothaman S., Galuppo V., Iaconetti C., Waring C.D. (2011). Endogenous cardiac stem cell activation by insulin-like growth factor-1/hepatocyte growth factor intracoronary injection fosters survival and regeneration of the infarcted pig heart. J. Am. Coll. Cardiol..

[24] Wang Y., Liu J., Tao Z., Wu P., Cheng W., Du Y., Zhou N., Ge Y., Yang Z. (2016). Exogenous HGF Prevents Cardiomyocytes from Apoptosis after Hypoxia via Up-Regulating Cell Autophagy. Cell. Physiol. Biochem..

[25] Kivelä R., Bry M., Robciuc M.R., Räsänen M., Taavitsainen M., Silvola J.M., Saraste A., Hulmi J.J., Anisimov A., Mäyränpää M.I. (2014). VEGF-B-induced vascular growth leads to metabolic reprogramming and ischemia resistance in the heart. EMBO Mol. Med..

[26] Gallo S., Sala V., Gatti S., Crepaldi T. (2015). Cellular and molecular mechanisms of HGF/Met in the cardiovascular system. Clin. Sci..

[27] Parizadeh S.M., Jafarzadeh-Esfehani R., Ghandehari M., Parizadeh M.R., Ferns G.A., Avan A., Hassanian S.M. (2019). Stem cell therapy: A novel approach for myocardial infarction. J. Cell. Physiol..

[28] Wernly B., Mirna M., Rezar R., Prodinger C., Jung C., Podesser B.K., Kiss A., Hoppe U.C., Lichtenauer M. (2019). Regenerative Cardiovascular Therapies: Stem Cells and Beyond. Int. J. Mol. Sci..

[29] Halappa N.G., Thirthalli J., Varambally S., Rao M., Christopher R., Nanjundaiah G.B. (2018). Improvement in neurocognitive functions and serum brain-derived neurotrophic factor levels in patients with depression treated with antidepressants and yoga. Indian J Psychiatry.

[30] Rodier M., Quirié A., Prigent-Tessier A., Béjot Y., Jacquin A., Mossiat C., Marie C., Garnier P. (2015). Relevance of Post-Stroke Circulating BDNF Levels as a Prognostic Biomarker of Stroke Outcome. Impact of rt-PA Treatment. PLoS ONE..

[31] Zhang Y., Zhang S.W., Khandekar N., Tong S.F., Yang H.Q., Wang W.R., Huang X.F., Song Z.Y., Lin S. (2017). Reduced serum levels of oestradiol and brain derived neurotrophic factor in both diabetic women and HFD-feeding female mice. Endocrine.

[32] Pius-Sadowska E., Machaliński B. (2017). BDNF—A key player in cardiovascular system. J. Mol. Cell. Cardiol..

[33] Fujimura H., Altar C.A., Chen R., Nakamura T., Nakahashi T., Kambayashi J., Sun B., Tandon N.N. (2002). Brain-derived neurotrophic factor is stored in human platelets and released by agonist stimulation. Thromb. Haemost..

[34] Nofuji Y., Suwa M., Sasaki H., Ichimiya A., Nishichi R., Kumagai S. (2012). Different circulating brain-derived neurotrophic factor responses to acute exercise between physically active and sedentary subjects. J. Sports Sci. Med..

[35] Araki S., Yamamoto Y., Dobashi K., Asayama K., Kusuhara K. (2014). Decreased plasma levels of brain-derived neurotrophic factor and its relationship with obesity and birth weight in obese Japanese children. Obes. Res. Clin. Pract..

[36] Lommatzsch M., Niewerth A., Klotz J., Schulte-Herbrüggen O., Zingler C., Schuff-Werner P., Virchow J.C. (2007). Platelet and plasma BDNF in lower respiratory tract infections of the adult. Respir. Med..

[37] Manni L., Nikolova V., Vyagova D., Chaldakov G.N., Aloe L. (2005). Reduced plasma levels of NGF and BDNF in patients with acute coronary syndromes. Int. J. Cardiol..

[38] Kermani P., Rafii D., Jin D.K., Whitlock P., Schaffer W., Chiang A., Vincent L., Friedrich M., Shido K., Hackett N.R. (2005). Neurotrophins promote revascularization by local recruitment of TrkB+ endothelial cells and systemic mobilization of hematopoietic progenitors. J. Clin. Investig..

[39] Halade G.V., Ma Y., Ramirez T.A., Zhang J., Dai Q., Hensler J.G., Lopez E.F., Ghasemi O., Jin Y.F., Lindsey M.L. (2013). Reduced BDNF attenuates inflammation and angiogenesis to improve survival and cardiac function following myocardial infarction in mice. Am. J. Physiol. Heart Circ. Physiol..

[40] Marotta P., Cianflone E., Aquila I., Vicinanza C., Scalise M., Marino F., Mancuso T., Torella M., Indolfi C., Torella D. (2018). Combining cell and gene therapy to advance cardiac regeneration. Expert Opin. Biol. Ther..

[41] Gnecchi M., He H., Liang O.D., Melo L.G., Morello F., Mu H., Noiseux N., Zhang L., Pratt R.E., Ingwall J.S. (2005). Paracrine action accounts for marked protection of ischemic heart by Akt-modified mesenchymal stem cells. Nat. Med..

[42] Gnecchi M., He H., Noiseux N., Liang O.D., Zhang L., Morello F., Mu H., Melo L.G., Pratt R.E., Ingwall J.S. (2006). Evidence supporting paracrine hypothesis for Akt-modified mesenchymal stem cell-mediated cardiac protection and functional improvement. FASEB J..

[43] Shafei A.E., Ali M.A., Ghanem H.G., Shehata A.I., Abdelgawad A.A., Handal H.R., Talaat K.A., Ashaal A.E., El-Shal A.S. (2017). Mesenchymal stem cell therapy: A promising cell-based therapy for treatment of myocardial infarction. J. Gene Med..

[44] Li H., Zuo S., He Z., Yang Y., Pasha Z., Wang Y., Xu M. (2010). Paracrine factors released by GATA-4 overexpressed mesenchymal stem cells increase angiogenesis and cell survival. Am. J. Physiol. Heart Circ. Physiol..

[45] Burchfield J.S., Dimmeler S. (2008). Role of paracrine factors in stem and progenitor cell mediated cardiac repair and tissue fibrosis. Fibrogenesis Tissue Repair.

[46] Okada S., Yokoyama M., Toko H., Tateno K., Moriya J., Shimizu I., Nojima A., Ito T., Yoshida Y., Kobayashi Y. (2012). Brain-derived neurotrophic factor protects against cardiac dysfunction after myocardial infarction via a central nervous system-mediated pathway. Arterioscler. Thromb. Vasc. Biol..

[47] Hang P., Zhao J., Cai B., Tian S., Huang W., Guo J., Sun C., Li Y., Du Z. (2015). Brain-derived neurotrophic factor regulates TRPC3/6 channels and protects against myocardial infarction in rodents. Int. J. Biol. Sci..

[48] Hang P., Sun C., Guo J., Zhao J., Du Z. (2016). BDNF-mediates Down-regulation of MicroRNA-195 Inhibits Ischemic Cardiac Apoptosis in Rats. Int. J. Biol. Sci..

[49] Miwa K., Lee J.K., Takagishi Y., Opthof T., Fu X., Hirabayashi M., Watabe K., Jimbo Y., Kodama I., Komuro I. (2013). Axon guidance of sympathetic neurons to cardiomyocytes by glial cell line-derived neurotrophic factor (GDNF). PLoS ONE.

[50] Ishida H., Saba R., Kokkinopoulos I., Hashimoto M., Yamaguchi O., Nowotschin S., Shiraishi M., Ruchaya P., Miller D., Harmer S. (2016). GFRA2 Identifies Cardiac Progenitors and Mediates Cardiomyocyte Differentiation in a RET-Independent Signaling Pathway. Cell Rep..

[51] Nakamura T., Mizuno S., Matsumoto K., Sawa Y., Matsuda H., Nakamura T. (2000). Myocardial protection from ischemia/reperfusion injury by endogenous and exogenous HGF. J. Clin. Investig..

[52] Shibuya M. (2011). Vascular Endothelial Growth Factor (VEGF) and Its Receptor (VEGFR) Signaling in Angiogenesis: A Crucial Target for Anti- and Pro-Angiogenic Therapies. Genes Cancer.

[53] Gavard J., Patel V., Gutkind J.S. (2008). Angiopoietin-1 prevents VEGF-induced endothelial permeability by sequestering Src through mDia. Dev. Cell.

[54] Jayasankar V., Woo Y.J., Pirolli T.J., Bish L.T., Berry M.F., Burdick J., Gardner T.J., Sweeney H.L. (2005). Induction of angiogenesis and inhibition of apoptosis by hepatocyte growth factor effectively treats postischemic heart failure. J. Card. Surg..

[55] Ono K., Matsumori A., Shioi T., Furukawa Y., Sasayama S. (1997). Enhanced expression of hepatocyte growth factor/c-Met by myocardial ischemia and reperfusion in a rat model. Circulation.

[56] Kardami E., Detillieux K., Ma X., Jiang Z., Santiago J.J., Jimenez S.K., Cattini P.A. (2007). Fibroblast growth factor-2 and cardioprotection. Heart Fail. Rev..

[57] Li Z., Guo X., Guan J. (2012). A thermosensitive hydrogel capable of releasing bFGF for enhanced differentiation of mesenchymal stem cell into cardiomyocyte-like cells under ischemic conditions. Biomacromolecules.

[58] Baumert B., Grymuła K., Pietruszka D., Kotowski M., Mielczarek M., Dziedziejko V., Hałasa M., Czerny B., Walczak M., Machaliński B. (2008). An optimization of hematopoietic stem and progenitor cell isolation for scientific and clinical purposes by the application of a new parameter determining the hematopoietic graft efficacy. Folia Histochem. Cytobiol..

